# Examining individual and geographic factors associated with social isolation and loneliness using Canadian Longitudinal Study on Aging (CLSA) data

**DOI:** 10.1371/journal.pone.0211143

**Published:** 2019-02-01

**Authors:** Verena H. Menec, Nancy E. Newall, Corey S. Mackenzie, Shahin Shooshtari, Scott Nowicki

**Affiliations:** 1 Department of Community Health Sciences, University of Manitoba, Manitoba, Canada; 2 Department of Psychology, Brandon University, Manitoba, Canada; 3 Department of Psychology, University of Manitoba, Manitoba, Canada; Indiana University Purdue University at Indianapolis, UNITED STATES

## Abstract

**Background:**

A large body of research shows that social isolation and loneliness have detrimental health consequences. Identifying individuals at risk of social isolation or loneliness is, therefore, important. The objective of this study was to examine personal (e.g., sex, income) and geographic (rural/urban and sociodemographic) factors and their association with social isolation and loneliness in a national sample of Canadians aged 45 to 85 years.

**Methods:**

The study involved cross-sectional analyses of baseline data from the Canadian Longitudinal Study on Aging that were linked to 2016 census data at the Forward Sortation Area (FSA) level. Multilevel logistic regression analyses were conducted to examine the association between personal factors and geographic factors and social isolation and loneliness for the total sample, and women and men, respectively.

**Results:**

The prevalence of social isolation and loneliness was 5.1% and 10.2%, respectively, but varied substantially across personal characteristics. Personal characteristics (age, sex, education, income, functional impairment, chronic diseases) were significantly related to both social isolation and loneliness, although some differences emerged in the direction of the relationships for the two measures. Associations also differed somewhat for women versus men. Associations between some geographic factors emerged for social isolation, but not loneliness. Living in an urban core was related to increased odds of social isolation, an effect that was no longer significant when FSA-level factors were controlled for. FSAs with a higher percentage of 65+ year old residents with low income were consistently associated with higher odds of social isolation.

**Conclusion:**

The findings indicate that socially isolated individuals are, to some extent, clustered into areas with a high proportion of low-income older adults, suggesting that support and resources could be targeted at these areas. For loneliness, the focus may be less on where people live, but rather on personal characteristics that place individuals at risk.

## Introduction

A large body of research shows that social isolation and loneliness have detrimental health consequences [1–4]. For example, social isolation has been shown to be associated with an increased risk of coronary heart disease and stroke [5], dementia [6], and mortality [2]. Similarly, loneliness is associated with a wide range of physical and mental health outcomes, such as physiological measures like increased blood pressure and depressed immune system [7,8], reduced cognitive function [9] and mortality [3]. Both social isolation and loneliness are related to increased health care use [10–13]. That social isolation and loneliness are serious concerns is increasingly being recognized by policy makers. For example, in the United Kingdom, the “Campaign to End Loneliness” is tackling loneliness by providing service organizations with information on how to approach the issue [14].

Social isolation and loneliness are often discussed alongside each other; however, they are conceptually different. Social isolation is typically defined in terms of the objective availability of social contacts or frequency of contact with social network members [1]. In essence, social isolation relates to whether a person is alone or part of a social network [15], typically composed of family or friends, but also the broader community environment through engagement in social activities [16–20]. In contrast, loneliness, which is sometimes referred to as “perceived social isolation” [8], refers to the perception that intimate and social needs are not being met [1,21]. It can arise from the perceived discrepancy between the quantity of social relationships that a person has, versus what they want [22]. Thus, a person can be socially isolated, but not lonely and vice versa. In order to mitigate health risks, it has been recommended that both concepts should be considered in research [3]. Although both social isolation and loneliness can be conceptualized as lying on a continuum (e.g., a continuum ranging from social isolation to social participation [15]), cut-offs are commonly used to identify individuals who are socially isolated or lonely [17,23–28]. A cut-off approach is essential to determine the prevalence of social isolation and loneliness, as well as, from an intervention perspective, to identify individuals who are socially isolated or lonely.

Previous research shows that a substantial proportion of adults are socially isolated or lonely; anywhere from less than 10% to 50% of middle-aged and older adults have been identified as being socially isolated or lonely [23,24,29–32]. Prevalence estimates vary widely as there are no standard measurement tools, particularly to assess social isolation, and no standard cut-offs to identify individuals who are socially isolated or lonely. Given their high prevalence estimates (even when conservative definitions are used) and well-documented negative physical and mental health consequences [1–9], preventing social isolation and loneliness, and providing supports to those who are socially isolated or lonely is critical. A first step in this direction is to identify the factors that place individuals at risk of social isolation and loneliness. The present study aims to address this issue by focusing on both personal and geographic risk factors in a national sample of middle-aged and older Canadians. In doing so, this study can help to identify individuals who might benefit from clinical interventions designed to reduce social isolation or loneliness [33–36]. It may also help policy makers identify geographic areas most in need of resources to support at risk individuals.

### Factors associated with social isolation and loneliness

Numerous studies have focused on identifying factors that are related to social isolation and loneliness. *Personal characteristics* that increase the likelihood of being socially isolated or lonely include very old age, being single or widowed, living alone, having less education, and having low income or financial strains [37–39]. There is less consistency in results for sex differences. Women have been found to be more likely to be lonely than men in some studies, although this effect tends to disappear when other factors are controlled for in the analyses [37,38]. For example, older women are more likely to be widowed and live alone than older men, both factors linked to loneliness. Yet other studies show that men are more likely to be lonely and socially isolated than women [27,40]. Studies also suggest that the factors that predict social isolation and loneliness differ for women and men [27,41]. For example, mobility problems have been shown to predict loneliness for women, but not men [41]. These findings highlight the importance of considering possible sex and/or gender differences in analyses, as they may suggest different targets for interventions for men versus women.

Although social isolation and loneliness have negative health consequences [1–9], the relationship is likely reciprocal in nature. Numerous studies have examined whether health-related factors are also possible risk factors for social isolation or loneliness [265–28,^40^–^44^]. For example, using data collected at two-year intervals over an 8-year time span, Barlow et al. [42] demonstrated that whereas individuals with many chronic conditions at baseline showed an increase in loneliness over time, the level of loneliness remained low over the 8 years for individuals with few chronic conditions. These health effects may be due to reduced social contacts; for example, mobility problems may make visiting family or friends difficult or impossible, thus leading to social isolation or loneliness.

Although many studies have examined personal characteristics in relation to social isolation and loneliness, there is a relative paucity of research on whether *geographic factors* are related to social isolation or loneliness. One to date under-researched issue in this respect is whether there are urban/rural differences in social isolation and loneliness. One the one hand, one might hypothesize that rural areas might be associated with a greater likelihood of social isolation and loneliness, given sparsely populated regions, long distances, and lacking infrastructure, particularly lack of transportation options which can interfere with social participation [45,46]. Moreover, migration can result in families being separated [47,48], which may also lead to social isolation or loneliness. On the other hand, rural residents often have strong community connections [49,50], which may be advantageous in terms of reducing the likelihood of social isolation and loneliness.

Indeed, research findings regarding urban/rural differences in social isolation and loneliness have been inconsistent. Whereas some studies indicate that rates of loneliness and social isolation are higher in urban, relative to more rural areas [28,51,52], others show greater loneliness in rural areas in bivariate analyses [53,54] or no urban/rural differences when controlling for other factors [26,54]. Moreover, studies show that the factors related to loneliness or social isolation are different for people living in rural versus urban areas [28,55]. A recent study that focused on loneliness in one metropolitan area further showed that the likelihood of loneliness was higher in areas further away from the city centre, relative to the city centre [56], suggesting that there are differences even within urban centres. Overall, the inconsistency in findings suggests the need for further research on possible urban/rural differences in social isolation and loneliness, including the need to examine different types of urban areas.

The sociodemographic characteristics of the place where people live also warrant examining. That such characteristics, particularly socioeconomic disparities, impact health has long been known and has been researched extensively [57]. However, there is a paucity of research on whether such factors relate to social isolation and loneliness specifically. One study that examined this issue showed that older individuals in financially more deprived neighborhoods in England were lonelier that their counterparts in financially better off neighborhoods [58].

In sum, although previous research has examined risk factors of social isolation and loneliness, studies have predominantly focused on personal characteristics. The present study contributes to the existing literature by examining not only personal characteristics, but also the role of geographic factors, including urban/rural differences and area-level sociodemographic differences, in social isolation and loneliness. The use of a national Canadian dataset allowed us to capture a wide variation in sociodemographic characteristics across different areas. Examining geographic factors is useful as it may identify whether socially isolated or lonely individuals cluster into certain geographic areas (or neighborhoods). As such, it can help policy makers and services providers target resources at specific areas [14], such as enhancing opportunities for social participation, transportation, or adapting the built environment to foster social participation (e.g., increasing neighborhood walkability). Thus, the main objective of the present study was to examine the relationship between personal (e.g., sex, income, health) and geographic (e.g., rural/urban; socio-demographic composition) factors and social isolation and loneliness among middle-aged and older Canadians. Moreover, we examined whether similar risk factors would emerge for women versus men, consistent with previous research that showed that the predictors of loneliness differed for women and men [27,41].

## Methods

### Data sources

This study involved a cross-sectional analysis of baseline data from the Canadian Longitudinal Study on Aging (CLSA) [59,60]. Baseline data were used, as the CLSA has only recently been launched and follow-up data were, at the time the present study was conducted, not yet available. CLSA consists of two cohorts: The Comprehensive Cohort involves participants who were randomly selected within age/sex strata from among individuals residing within 25 km of a data collection site (or 50 km in lower density cities) in ten sites across Canada. These participants were interviewed in their own homes with computer-assisted interview instruments. They also came to data collection sites for additional computer-assisted interviews and comprehensive assessments (e.g., physical measures, biological samples). The Tracking Cohort consists of a randomly (within age/sex strata) selected sample from the 10 Canadian provinces that completed computer-assisted telephone interviews. Participant exclusion criteria were: could not communicate in one of the two national languages, English or French; cognitive impairment at time of contact; resident of the three territories; full-time member of the Canadian Armed Forces; resident in a long-term care institution; and living on Federal First Nations reserves or other First Nations settlements. CLSA participants provided written consent before participating in the study.

Public access census data from 2016 were used to derive geographic variables. CLSA questionnaire data were linked to census data via the first three digits of participants’ postal code (Forward Sortation Area, FSA). Postal codes are used by Canada Post for the purpose of sorting and delivering mail, with FSAs representing geographic areas. As of 2011, there were 1638 FSAs in Canada [61].

The present study received ethics approval from the University of Manitoba’s Health Research Ethics Board.

### Study sample

A total of 51,338 participants living in 1558 FSAs participated in the CLSA baseline. Of these, FSAs with less than 10 participants were excluded from analyses, as estimates of social isolation and loneliness are less meaningful for these FSAs. Our final sample included 48,330 participants aged 45 to 85 from 977 FSAs. Because of missing values on some variables, the unweighted sample size in the analyses for social isolation was 47,752; for loneliness the final sample size was 47,818. A comparison of FSAs that were included in the analyses to those excluded indicated that they were comparable (e.g., 51.0% versus 50.4% for the % women in the FSAs). CLSA participants were also similar. For example, 51% of CLSA participants in the included FSAs were female and 5.6% had a household income of less than $20,000, compared to 50.2% and 6.3%, respectively in the excluded FSAs.

### Measures

#### Social isolation

Social isolation has been defined in different ways in previous literature [1]. Conceptually, our definition was guided by the Convoy Model of Social Relationships, according to which individuals are surrounded by a series of social network ties that range from closest to less close [62]. Spouses tend to play a key role in people’s social networks, followed by children, siblings (or other relatives), and friends. Consistent with the finding that a variety of social network members play an important role in people’s lives [19,20], contact with these network members has been used to define social isolation in previous research [16–18].

A social isolation index was derived based on five sets of questions: 1) marital status (married or living in a common-law relationship; never married or never lived with a partner; divorced; separated; widowed); 2) living arrangements (number of people currently living in household); 3) when participants last got together with each of the following social network members living outside of their household: children, siblings, close friends, and neighbors (1 = within the last day or two; 2 = within the last week or two; 3 = within the past month; 4 = within the past 6 months; 5 = within the past year; 6 = more than 1 year ago); 4) retirement status (retired, working part-time/full-time); and, social participation in eight activities in the past 12 months (e.g., family or friendship based activities, church or religious activities, sports or physical activities, and educational and cultural activities; 1 = at least once a day; 2 = at least once a week; 3 = at least once a month; 4 = at least once a year; 5 = never)(see https://datapreview.clsa-elcv.ca/ for the entire questionnaire). The social participation questions were recoded such that response categories 4 and 5 were coded as 0 (i.e. less than once a month), and response categories 1–3 were coded as 1 (i.e. at least once a month or more often). The re-coded variables were then summed to create a new score ranging from 0–8.

Similar to previous research [16–18], we allocated one point when each of the following conditions applied: 1) living alone and not married or in a common law relationship; 2) got together with friends or neighbours “within the past 6 months” or less frequently, or reported having no friends or neighbors; 3) got together with relatives/siblings “within the past 6 months” or less frequently, or reported having no relatives or siblings; 4) got together with children “within the past 6 months” or less frequently, or had no children. A fifth criterion was that one point was allocated if participants were retired and had little social participation (scores 0 or 1 on our re-coded social participation scale; i.e. they participated in none or only one of the activities at least once a month or more often). This resulted in a social isolation index ranging from 0–5, with higher scores reflecting greater social isolation.

As we were interested in identifying socially isolated groups of adults and their associated characteristics, we subsequently dichotomized the social isolation index. There are no established cut-offs in the literature to define individuals who are socially isolated. For the present purposes, we classified individuals with scores 3–5 on the index as socially isolated (coded as 1) and those with scores 0–2 as not socially isolated (coded as 0). This cut-off was chosen, as it classifies people with at least half of the criteria that make up the social isolation index as being socially isolated. As contact with social network members is only measured in terms of in-person contact in the CLSA, the cut-off, in part, also ensures that individuals who may have had contact with some social network members via other means only (e.g., telephone, internet) are not identified as being socially isolated. For example, individuals with no family (relatives/siblings, children) living close enough to allow frequent direct contact would not be considered socially isolated, unless they also met one other criterion. Sensitivity analyses were also conducted with a 2–5 (vs. 0/1) cut-off.

Given that CLSA contains two cohorts (Tracking and Comprehensive) we compared the percent socially isolated in each cohort. The (unweighted) percentage of participants identified as socially isolated using a 3+ cut-off on our social isolation index was relatively similar in the Tracking cohort, compared to the Comprehensive cohort (6.7% vs. 5.5%).

#### Loneliness

As the CLSA baseline questionnaire does not contain a loneliness scale, a single-item loneliness question that is part of the CESD depression scale [63] was used. Questions focus on the past week and participant were asked: “How often did you feel lonely?” (1 = all of the time [5-7days]; 2 = occasionally [3–4 days]; 3 = some of the time [1–2 days]; 4 = rarely or never [less than 1 day]. Similar single-item measures are commonly used in the literature [1]. The item was dichotomized, with “all of the time” and “occasionally” responses considered lonely (coded as 1) and the remaining categories as not lonely (coded as 0). The (unweighted) percent of participants classified as being lonely was similar in the Tracking versus Comprehensive cohort (11.2% vs. 10.8%).

#### Personal factors

Personal factors included: age, sex, education, household income, functional status, and chronic conditions. Age was categorized into four categories (ages 45–54, 55–64, 65–74, and 75–85). Sex was coded as 0 = women and 1 = men. Education was dichotomized as: 0 = secondary school or less, and 1 = at least some post-secondary education. Household income was included as an overall measure of the financial resources available to an individual. It was measured by asking participants to give the best estimate of the total household income received by all household members, from all sources, before taxes and deductions, in the past 12 months (1 = less than $20,000; 2 = $20,000 or more, but less than $50,000; 3 = $50,000 or more, but less than $100,000; 4 = $100,000 or more, but less than $150,000; and 5 = $150,000 or more). As a considerable number of participants did not answer the question, a “missing” category was also included in order not to lose these individuals from analyses. This also allowed us to determine if individuals who did not answer the question differed systematically from those who did.

Functional status was assessed using the Older Americans’ Resources and Services (OARS) Multidimensional Functional Assessment Questionnaire [64]. The scale includes seven questions related to Activities of Daily Living (e.g., getting out of bed, dressing, and eating) and seven questions related to Instrumental Activities of Daily Living (e.g., using the telephone, shopping, and preparing meals). For each question, participants responded whether they can complete the task without help, with some help, or are completely unable to perform it. The items can be used to categorize individuals into: no functional impairment; mild impairment; moderate impairment; severe impairment; and total impairment. As most participants had no functional impairment, responses were dichotomized: 0 = no functional impairment”, and 1 = at least some functional impairment.

Chronic conditions were measured with a list of 33 conditions, such as osteoarthritis, respiratory conditions, and cardiac/cardiovascular conditions, with participants asked if a doctor had diagnosed them with the condition. An index was created by summing affirmative responses.

#### Geographic factors

A rural/urban variable is available in the CLSA data. The variable was added by CLSA based on a Statistics Canada definition and postal code conversion file [65,66]. “Urban core” refers to census metropolitan areas (CMA) with a population of at least 100,000 (50,000 or more of which live in the core) or census agglomerations (CA) with a core population of at least 10,000. “Secondary core” refers to a population center within a CMA with at least 10,000 residents that was the core of a CA but has now been merged with an adjacent CMA. Urban and secondary cores were combined in the present study. “Urban fringe” refers to population centers within a CMA or CA that are not contiguous with the core or secondary core with fewer than 10,000 residents. “Urban population centres outside CMA and CA” are defined as settlements outside CMA and CA with a population of at least 1,000 and a population density of 400 persons or more per square kilometer. “Rural” is defined as areas within CMA or CA not classified as core or fringe, or areas not defined as population centres. A “not defined” category was also included for which no urban/rural information was available. Although the rural/urban variable was considered a geographic variable in this study, it should be noted that it was not measured at the FSA-level, unlike the other geographic variables. FSAs are not necessarily aligned with the Statistics Canada rural/urban definition. This means that some FSAs contain both rural and urban areas, as defined by Statistics Canada. In the analyses, rural/urban was treated as an individual-level variable.

Other geographic-based variables were derived from 2016 public access census data for each FSA. We selected census variables that were similar to individual predictors of social isolation and loneliness, including: percent of women; percent of the population aged 65+; and percent of the population living alone. Census data further contain the percent of the population aged 65 or older with low income based on the after-tax low-income cut-offs (LICO-AT). Statistics Canada defines the LICO-AT as an after-tax income threshold below which a family is expected to spend a larger share of its income on food, shelter and clothing (20 percentage points more) than the average family [67]. The percent of the population whose first language was not one of the two official languages (English, French), was also included as a way to assess the ethnic diversity of areas.

#### Analyses

Data were analyzed using multilevel analyses, given the nested nature of the data (individuals within FSA) using SAS version 9.4. Given that social isolation and loneliness were defined as dichotomous outcomes, logistic regressions were conducted using Proc Glimmix.

A series of analyses were conducted for the two outcome variables. First, in model 1, variables measured at the individual-level were entered into the regression model. This included personal characteristics, as well as the rural/urban variable which, as noted above, was not available at the FSA level; second, in model 2, variables measured at the FSA-level were added. This approach was taken for both the full sample and, subsequently, for women and men, respectively. For loneliness, all variables were included in the analyses; for social isolation, variables that were included in its definition (marital status and living alone) were excluded in the analyses, as was the FSA-level variable percent living alone, given that it was strongly correlated with living alone. Effects were evaluated for significance at p < .01.

## Results

Sample characteristics are displayed in Table 1.

**Table 1:** Sample description.

Measures	% or Mean (SE)^a^
**Age groups**	
45–54 years old	37.19 (0.38)
55–64 years old	31.25 (0.34)
65–74 years old	19.36 (0.28)
75–85 years old	12.19 (0.20)
**Sex**	
Women	51.48 (0.38)
Men	48.52 (0.38)
**Marital status**	
Never married/never lived with a partner	7.88 (0.19)
Widowed	7.23 (0.16)
Divorced	7.81 (0.17)
Separated	2.37 (0.11)
Married/common-law partnership	74.71 (0.30)
**Living arrangements**	
Living alone	17.11 (0.24)
Living with somebody	82.89 (0.24)
**Education**	
Less than postsecondary	39.57 (0.36)
Postsecondary	60.43 (0.36)
**Household income (yearly)**	
< $20,000	4.93 (0.14)
$20,000 to < $50,000	22.26 (0.30)
$50,000 to < $100,000	33.84 (0.36)
$100,000 to < $150,000	18.51 (0.30)
> = $150,000	15.03 (0.28)
Missing response	5.43 (0.15)
**Functional status**	
No functional impairment	90.87 (0.20)
Mild, moderate, severe, total impairment	9.13 (0.20)
**Number of chronic diseases**^b^	3.05 (0.02)
**Location of residence**	
Rural	21.76 (0.33)
Urban core	65.69 (0.36)
Urban fringe	1.58 (0.1)
Urban outside CMA and CA^c^	5.54 (0.17)
Not defined	5.43 (0.14)

^a^ Weighted means and their standard errors are shown.

^b^ Sum of 33 summed chronic conditions.

^c^ CMA refers to census metropolitan area; CA refers to census agglomeration.

Table 2 shows descriptive statistics for FSA-level measures. There was considerable variation across the 977 FSAs. For example, the percent individuals aged 65+ ranged from 4% to 40.2%.

**Table 2:** Descriptive statistics: FSA-level measures (N = 977).

Measures	% (SE)	Minimum	Maximum
% Women	51.04 (1.54)	42.30	56.00
% Age 65+	18.05 (5.37)	4.00	40.20
% Who speak non-official language^a^	17.60 (16.04)	0.19	75.44
% 65+ with low income	5.44 (6.06)	0	51.00
% Living alone	28.94 (9.90)	8.48	66.77

Note. FSA = Forward Sortation Area; ^a^ languages other than English and French

The prevalence of social isolation and loneliness by personal characteristics is shown in Table 3. Overall, the prevalence of social isolation was 5.1%, but varied widely across personal characteristics.

**Table 3:** Personal characteristics by social isolation and loneliness.

	Socially isolated (unweighted N = 47,752)			Lonely (unweighted N = 47,818)		
	Total sample	Women	Men	Total sample	Women	Men
Measures	% (SE)	% (SE)	% (SE)	% (SE)	% (SE)	% (SE)
**Overall**	5.09 (0.15)	4.78 (0.20)	5.41 (0.22)	10.20 (0.21)	11.11 (0.31)	9.24 (0.30)
**Age groups**						
45–54 years old	3.24 (0.22)	2.50 (0.27)	3.98 (0.34)	8.87 (0.37)	8.78 (0.52)	8.97 (0.53)
55–64 years old	5.08 (0.27)	4.71 (0.37)	5.46 (0.40)	10.56 (0.39)	11.48 (0.57)	9.60 (0.52)
65–74 years old	6.28 (0.35)	5.91 (0.47)	6.66 (0.53)	10.36 (0.46)	11.88 (0.67)	8.73 (0.64)
75–85 years old	8.86 (0.47)	9.57 (0.69)	8.00 (0.63)	13.10 (0.56)	15.58 (0.84)	10.02 (0.67)
**Sex**						
Women	4.78 (0.20)	-	-	11.11 (0.31)	-	-
Men	5.41 (0.22)	-	-	9.24 (0.30)	-	-
**Marital status**						
Never married/never lived with a partner	-	-	-	18.59 (0.96)	15.55 (1.24)	22.03 (1.48)
Widowed	-	-	-	24.78 (0.98)	22.44 (1.07)	33.68 (2.27)
Divorced	-	-	-	18.00 (0.87)	15.42 (0.94)	22.75 (1.74)
Separated	-	-	-	24.41 (2.07)	22.79 (2.9)	26.19 (2.95)
Married/common-law partnership	-	-	-	6.65 (0.22)	7.73 (0.35)	5.69 (0.27)
**Living arrangements**						
Living alone	-	-	-	23.07 (0.63)	20.47 (0.74)	27.89 (1.16)
Living with somebody	-	-	-	7.55 (0.22)	8.55 (0.33)	6.61 (0.29)
**Education**						
Less than postsecondary	6.47 (0.27)	5.90 (0.39)	7.04 (0.39)	12.04 (0.37)	13.19 (0.54)	10.87 (0.50)
Postsecondary	4.18 (0.17)	4.07 (0.22)	4.30 (0.25)	9.00 (0.26)	9.78 (0.37)	8.15 (0.37)
**Household income (yearly)**						
< $20,000	23.48 (1.25)	18.28 (1.44)	32.70 (2.31)	24.93 (1.25)	22.14 (1.45)	29.96 (2.29)
$20,000 to < $50,000	8.06 (0.38)	7.31 (0.48)	9.12 (0.62)	14.69 (0.53)	15.72 (0.71)	13.25 (0.78)
$50,000 to < $100,000	3.67 (0.23)	3.04 (0.31)	4.28 (0.34)	9.34 (0.37)	9.49 (0.53)	9.19 (0.52)
$100,000 to < $150,000	1.19 (0.15)	0.99 (0.2)	1.38 (0.23)	5.55 (0.40)	6.05 (0.59)	5.10 (0.54)
> = $150,000	1.19 (0.24)	0.90 (0.29)	1.41 (0.36)	4.93 (0.40)	5.94 (0.72)	4.19 (0.45)
Missing response	9.38 (0.79)	8.09 (0.91)	11.93 (1.52)	14.39 (0.97)	13.68 (1.16)	15.80 (1.77)
**Functional status**						
No functional impairment	4.49 (0.15)	4.15 (0.2)	4.82 (0.21)	9.19 (0.22)	9.83 (0.32)	8.57 (0.30)
Mild, moderate, severe, total impairment	10.43 (0.68)	8.33 (0.72)	15.84 (1.56)	19.29 (0.90)	18.71 (1.04)	20.78 (1.79)
**Location of residence**						
Rural	4.60 (0.35)	3.81 (0.46)	5.50 (0.54)	9.49 (0.51)	9.94 (0.73)	8.98 (0.72)
Urban core	5.20 (0.18)	5.14 (0.25)	5.26 (0.25)	10.49 (0.26)	11.73 (0.38)	9.23 (0.35)
Urban fringe	2.80 (0.75)	3.32 (1.23)	2.32 (0.88)	10.27 (1.79)	9.24 (2.24)	11.26 (2.78)
Urban outside CMA and CA^a^	6.45 (0.8)	5.38 (0.85)	7.79 (1.44)	9.51 (0.88)	9.90 (1.19)	9.03 (1.33)
Not defined	4.89 (0.51)	4.25 (0.66)	5.67 (0.78)	10.32 (0.81)	10.55 (1.14)	10.04 (1.13)

This table shows weighted row percentages. Marital status and living arrangements are not shown for social isolation as they were part of its operational definition.

^a^ CMA refers to census metropolitan area; CA refers to census agglomeration.

Correlations between geographically defined (FSA-level) measures and social isolation and loneliness are shown in Table 4. Weak, but statistically significant correlations emerged between all measures and social isolation, except for the percent women. For loneliness, weak correlations emerged between the percent 65+ with low income and the percent living alone.

**Table 4:** Correlations between geographic measures and social isolation and loneliness.

FSA-level measures	Social isolation	Loneliness
% Women	0.003	-0.004
% Age 65+	0.015**	0.005
% Who speak non-official language^a^	0.015**	-0.004
% 65+ with low income	0.058**	0.019**
% Living alone	-	0.040**

This table shows Spearman correlation coefficients.

* p < .01;

** p < .001.

^a^ languages other than English and French.

Results of the regression models for social isolation are provided in Table 5. In the final model (model 2), for the total sample, being 45–54 years old was associated with reduced odds of social isolation, relative to being 75–85 years old. Having less than postsecondary education, and a household income higher than $20,000 (or not responding to the household income question) was also associated with reduced odds of social isolation. Conversely, being male, having functional impairment, and more chronic conditions was associated with increased odds of being socially isolated. Moreover, the higher the percent 65+, and percent 65+ with low income in an FSA, the higher the odds of being socially isolated. Noteworthy is that whereas living in an urban core was related to increased odds of social isolation in model 1 without FSA-level variables for both the total sample and women (adjusted odds ratio, AOR = 1.36 and 1.55, respectively), this was no longer the case when FSA-level variables were added in model 2. In model 2, a higher percent 65+ with low income (AOR = 1.04, p < .001) and higher percent 65+ in general (AOR = 1.02; p < .01) were associated with increased odds of social isolation.

**Table 5:** Multilevel regression results for social isolation (unweighted N = 47,752).

Measures	Model 1			Model 2		
	Adjusted odds ratios (99% CI)			Adjusted odds ratios (99% CI)		
	Total sample	Women	Men	Total sample	Women	Men
45–54 years vs. 75–85	0.75 (0.63,1.90)	0.59 (0.45,0.77)	0.87 (0.68,1.11)	0.75 (0.62,0.89)	0.59 (0.45,0.76)	0.85 (0.67,1.10)
55–64 years vs. 75–85	0.92 (0.79,1.06)	0.83 (0.68,1.02)	0.95 (0.77,1.18)	0.91 (0.79,1.06)	0.83 (0.68,1.02)	0.94 (0.76,1.17)
65–74 years vs. 75–85	0.87 (0.76,1.01)	0.80 (0.66,0.98)	0.93 (0.75,1.15)	0.87 (0.76,1.01)	0.80 (0.66,0.98)	0.93 (0.75,1.15)
Men vs. women	1.44 (1.30,1.61)	-	-	1.43 (1.29,1.60)	-	-
Less than postsecondary education vs. postsecondary	0.79 (0.70,0.88)	0.69 (0.59,0.81)	0.89 (0.76,1.04)	0.80 (0.71,0.89)	0.70 (0.60,0.82)	0.91 (0.78,1.07)
$20,000 to < $50,000 vs. < $20,000	0.34 (0.29,0.40)	0.43 (0.34,0.53)	0.24 (0.19,0.31)	0.35 (0.30,0.41)	0.44 (0.35,0.54)	0.26 (0.20,0.32)
$50,000 to < $100,000 vs. < $20,000	0.15 (0.12,0.17)	0.20 (0.16,0.26)	0.10 (0.08,0.13)	0.16 (0.13,0.18)	0.21 (0.17,0.27)	0.11 (0.09,0.14)
$100,000 to < $150,000 vs. < $20,000	0.06 (0.05,0.08)	0.07 (0.05,0.11)	0.04 (0.03,0.06)	0.06 (0.05,0.08)	0.08 (0.05,0.12)	0.05 (0.03,0.07)
> = $150,000 vs. < $20,000	0.04 (0.03,0.06)	0.07 (0.05,0.12)	0.03 (0.02,0.04)	0.05 (0.03,0.07)	0.08 (0.05,0.13)	0.03 (0.02,0.05)
Missing response vs. < $20,000	0.33 (0.27,0.41)	0.39 (0.29,0.52)	0.26 (0.19,0.36)	0.35 (0.29,0.44)	0.41 (0.31,0.55)	0.28 (0.20,0.39)
Mild, moderate, severe, total impairment vs. no impairment	1.35 (1.16,1.57)	1.19 (0.98,1.45)	1.65 (1.29,2.10)	1.35 (1.16,1.57)	1.19 (0.98,1.44)	1.65 (1.29,2.10)
Number of chronic conditions	1.02 (1.00,1.05)	1.04 (1.01,1.07)	1.01 (0.98,1.04)	1.02 (1.00,1.05)	1.04 (1.01,1.07)	1.01 (0.98,1.04)
Urban core vs. rural	1.36 (1.11,1.67)	1.55 (1.16,2.06)	1.25 (0.96,1.63)	1.20 (0.96,1.50)	1.26 (0.92,1.73)	1.16 (0.86,1.57)
Urban fringe vs. rural	0.83 (0.46,1.52)	0.89 (0.39,2.03)	0.81 (0.36,1.81)	0.91 (0.51,1.64)	0.94 (0.41,2.12)	0.92 (0.41,2.03)
Urban outside CMA and CA vs. rural	1.14 (0.82,1.59)	1.37 (0.87,2.15)	1.01 (0.63,1.62)	1.17 (0.84,1.61)	1.38 (0.88,2.15)	1.04 (0.65,1.66)
Not defined vs. rural	1.11 (0.81,1.51)	1.22 (0.78,1.90)	1.03 (0.67,1.57)	1.09 (0.80,1.49)	1.18 (0.76,1.83)	1.04 (0.68,1.58)
% Women	-	-	-	0.98 (0.93,1.04)	1.02 (0.95,1.10)	0.95 (0.89,1.02)
% Age 65+	-	-	-	1.02 (1.00,1.03)	1.02 (1.00,1.04)	1.02 (1.00,1.04)
% Who speak non-official language^a^	-	-	-	1.00 (0.99,1.00)	1.00 (0.99,1.01)	1.00 (0.99,1.00)
% 65+ with low income	-	-	-	1.04 (1.03,1.06)	1.05 (1.03,1.07)	1.04 (1.03,1.06)

Results are presented from multilevel logistic regressions conducted for the total sample and women and men, respectively. Model 1 included only individual-level variables. In Model 2, area-level (FSA-level) variables were added.

^a^ Languages other than English and French.

Results were somewhat different for women versus men. Among women, older age, higher education, low income, more chronic conditions, and a higher percent 65+ with low income were all significantly associated with increased odds of social isolation in model 2. Among men, only low income, functional impairment, and a higher percent 65+ with low income were associated with higher odds of social isolation.

Results were also similar in the sensitivity analyses for which we defined social isolation in terms of a 2+ cut-off on our social isolation index. Again, all personal characteristics were significantly associated with social isolation. Among the geographic factors, only the percent 65+ and the percent 65+ with low income were associated with social isolation.

For loneliness (see Table 6), significant associations only emerged for personal characteristics. Moreover, results were similar for women and men. Both younger women and men reported being more lonely than their oldest counterparts. Not being married or in a common-law relationship, living alone, having a household income of less than $20,000, functional impairment, and more chronic conditions all increased the odds of being lonely.

**Table 6:** Multilevel regression results for loneliness (unweighted N = 47,818).

Measures	Model 1			Model 2		
	Adjusted odds ratios (99% CI)			Adjusted odds ratios (99% CI)		
	Total sample	Women	Men	Total sample	Women	Men
45–54 years vs. 75–85	1.64 (1.42,1.89)	1.47 (1.21,1.78)	1.75 (1.41,2.16)	1.63 (1.42,1.88)	1.46 (1.21,1.78)	1.75 (1.41,2.16)
55–64 years vs. 75–85	1.56 (1.38,1.76)	1.47 (1.25,1.73)	1.60 (1.32,1.93)	1.56 (1.38,1.76)	1.47 (1.24,1.73)	1.60 (1.32,1.93)
65–74 years vs. 75–85	1.12 (0.99,1.27)	1.11 (0.95,1.30)	1.11 (0.91,1.34)	1.12 (0.99,1.26)	1.11 (0.94,1.30)	1.11 (0.91,1.34)
Men vs. women	1.13 (1.04,1.22)	-	-	1.12 (1.03,1.22)	-	-
Never married/never lived with a partner vs. married	1.80 (1.54,2.12)	1.41 (1.14,1.75)	2.19 (1.72,2.80)	1.79 (1.52,2.10)	1.41 (1.13,1.75)	2.16 (1.69,2.77)
Divorced vs. married	1.84 (1.58,2.14)	1.42 (1.17,1.72)	2.53 (1.99,3.22)	1.83 (1.58,2.13)	1.41 (1.16,1.71)	2.52 (1.99,3.21)
Separated vs. married	2.31 (1.87,2.85)	1.74 (1.30,2.33)	2.97 (2.19,4.03)	2.30 (1.87,2.84)	1.73 (1.30,2.32)	2.97 (2.19,4.03)
Widowed vs. married	3.04 (2.61,3.54)	2.26 (1.87,2.74)	4.89 (3.81,6.27)	3.04 (2.61,3.54)	2.25 (1.86,2.73)	4.89 (3.81,6.28)
Living alone vs. with somebody	1.51 (1.33,1.71)	1.37 (1.17,1.61)	1.69 (1.38,2.06)	1.51 (1.33,1.71)	1.38 (1.17,1.62)	1.68 (1.37,2.05)
Less than postsecondary education vs. postsecondary	1.10 (1.01,1.19)	1.12 (1.00,1.25)	1.06 (0.93,1.20)	1.10 (1.01,1.20)	1.12 (1.00,1.25)	1.06 (0.93,1.21)
$20,000 to < $50,000 vs. < $20,000	0.84 (0.73,0.96)	0.90 (0.75,1.08)	0.72 (0.57,0.90)	0.84 (0.73,0.97)	0.90 (0.75,1.08)	0.72 (0.57,0.91)
$50,000 to < $100,000 vs. < $20,000	0.69 (0.59,0.80)	0.71 (0.58,0.86)	0.60 (0.48,0.77)	0.69 (0.59,0.80)	0.71 (0.58,0.86)	0.61 (0.48,0.78)
$100,000 to < $150,000 vs. < $20,000	0.50 (0.41,0.61)	0.50 (0.39,0.65)	0.46 (0.35,0.61)	0.50 (0.42,0.61)	0.50 (0.39,0.65)	0.47 (0.35,0.62)
> = $150,000 vs. < $20,000	0.47 (0.38,0.58)	0.48 (0.36,0.65)	0.43 (0.32,0.59)	0.47 (0.38,0.59)	0.48 (0.36,0.64)	0.44 (0.32,0.60)
Missing response vs. < $20,000	0.82 (0.68,0.98)	0.86 (0.68,1.08)	0.67 (0.49,0.92)	0.82 (0.68,0.99)	0.86 (0.68,1.09)	0.68 (0.50,0.93)
Mild, moderate, severe, total impairment vs. no impairment	1.42 (1.26,1.60)	1.37 (1.19,1.58)	1.51 (1.22,1.86)	1.42 (1.27,1.60)	1.37 (1.19,1.58)	1.51 (1.22,1.86)
Number of chronic conditions	1.07 (1.05,1.09)	1.07 (1.05,1.09)	1.08 (1.05,1.11)	1.07 (1.05,1.09)	1.07 (1.05,1.09)	1.08 (1.05,1.11)
Urban core vs. rural	1.02 (0.90,1.15)	1.05 (0.89,1.23)	1.03 (0.86,1.23)	1.02 (0.88,1.18)	1.04 (0.86,1.27)	1.02 (0.82,1.27)
Urban fringe vs. rural	1.13 (0.82,1.56)	1.06 (0.68,1.64)	1.26 (0.78,2.02)	1.14 (0.82,1.58)	1.05 (0.67,1.63)	1.28 (0.79,2.08)
Urban outside CMA and CA vs. rural	0.98 (0.78,1.23)	0.94 (0.69,1.27)	1.09 (0.77,1.55)	0.98 (0.78,1.24)	0.94 (0.70,1.28)	1.09 (0.77,1.56)
Not defined vs. rural	0.97 (0.79,1.20)	0.97 (0.73,1.28)	0.99 (0.71,1.36)	0.97 (0.79,1.20)	0.97 (0.73,1.29)	0.98 (0.71,1.36)
% Women	-	-	-	0.99 (0.95,1.02)	1.00 (0.95,1.04)	0.99 (0.94,1.04)
% Age 65+	-	-	-	1.00 (0.99,1.01)	1.00 (0.99,1.01)	1.00 (0.99,1.02)
% Who speak non-official language^a^	-	-	-	1.00 (1.00,1.00)	1.00 (0.99,1.00)	1.00 (0.99,1.01)
% 65+ with low income	-	-	-	1.01 (1.00,1.02)	1.01 (0.99,1.02)	1.01 (0.99,1.02)
% Living alone	-	-	-	1.00 (0.99,1.00)	1.00 (0.99,1.00)	1.00 (0.99,1.01)

Results are presented from multilevel logistic regressions conducted for the total sample and women and men, respectively. Model 1 included only individual-level variables. In Model 2, area-level (FSA-level) variables were added.

^a^ Languages other than English and French.

## Discussion

Several key findings emerged in the present study. Overall, using our definitions, the prevalence of social isolation and loneliness was 5.1% and 10.2%, respectively, but there was substantial variation across personal characteristics in prevalence rates. Being older, male, having a low income, functional impairment and more chronic conditions were all associated with increased odds of being socially isolated, as was, somewhat counter-intuitively, a higher education level. The finding for education is consistent with a Swedish study that showed that individuals with higher education, compared to those with less education had less dense local family networks, which may be due to migration patterns [47]. A similar issue may be at play in the present study in that more highly educated younger individuals, as compared to those with less education, may be more likely to move for job opportunities or, conversely, more highly educated older individuals may be more likely to move to retirement communities. Both scenarios would result in individuals having less direct contact with family members.

For loneliness, being younger, being male, living alone, and having a low education level, low income, functional impairment and more chronic conditions increased the odds of being lonely, consistent with previous research. [37,38]. In addition, marital status emerged as a major factor related to loneliness. While all non-partnered states emerged as a risk factor for loneliness, this was particularly the case for widowed men, for whom the odds of being lonely were more than four times higher than their married/common-law counterparts. Similar results for widowed men have been found in previous research [41]. These findings underscore the profound impact that losing a partner has [68]. For men, the impact of losing a spouse may be compounded by the relative lack of a diverse social network. In the present study, men were more likely to be socially isolated than women, indicative of a smaller social network with whom they have frequent contact, which suggests that they may not have other network ties that could, at least in part, compensate for the loss of a partner.

It is noteworthy that although the factors associated with social isolation and loneliness were similar for women and men, there were two exceptions, both related to age effects: whereas younger women aged 45–54 were less likely to be socially isolated than their older counterparts (those 75–85 years old), they were more likely to be lonely. For men, social isolation did not differ across age groups, yet younger men were more likely to be lonely than older men. On the one hand, these findings highlight that social isolation is not synonymous with loneliness, consistent with what has been argued in the literature [1,15,21], and that one can be lonely regardless of the frequency of contact with social network members. In this respect, a recent study with young adults (aged 21–30) and middle-aged adults (aged 50–70) shows that social network size, frequency of contact with network members, and social participation was not consistently related to loneliness [69]. On the other hand, our finding that older adults were less likely to be lonely than younger individuals may suggest that older adults may adapt to a shrinking social network, perhaps by adjusting their expectations of how frequent contact with social network members should be. Alternatively, as predicted by Socio-emotional Selectivity Theory, older adults may intentionally “prune” peripheral social network members in order to focus on close, emotionally meaningful relationships, as opposed to maintaining contacts that are less emotionally satisfying [70].

In terms of geographic factors, of interest was whether an urban/rural difference would emerge for social isolation and loneliness. Previous research as to whether individuals living in rural areas are more socially isolated and lonely than their counterparts in urban areas, or vice versa, has led to inconsistent findings [51–54]. In the present study, living in a large urban center, relative to a rural area, was associated with an increased likelihood of social isolation for the total sample and for women. However, this effect was no longer significant when FSA-level factors were included in the analyses. This may suggest that this effect is either weak or related to other factors that are correlated with urban centers, in particular, the percent 65+ year old residents with low income. In other words, it may be that living in a city was related to social isolation because cities are more likely than rural areas to have socioeconomically deprived neighborhoods. This interpretation is consistent with previous research that shows that social exclusion is clustered into socioeconomically deprived neighborhoods [58] and well as the literature on the negative health consequences associated with living in poor neighborhoods [57].

From an intervention perspective, the findings suggest that in order to reduce social isolation, support and resources could, to some extent, be targeted at certain areas, particularly city neighborhoods with a high proportion of older adults who live on low income. The concept of ‘age-friendly’ communities [71], which has been gaining increasing attention in the last decade on the part of policy makers is relevant in this respect. An age-friendly community or city (or neighborhood) provides supports in the physical and social environment, such as aspects of the housing environment (e.g. availability of affordable housing), public spaces (e.g., accessible buildings, walkability), and opportunities for social participation (e.g., availability of social programs for older adults) [71]. Making communities more age-friendly may enhance social connectivity [72], and may provide one approach to reducing social isolation.

The finding that geographic variables were not associated with loneliness again supports the view that loneliness is conceptually different from social isolation, reflective of the perception of whether one’s contact with other people is sufficient. The present findings suggest that these perceptions are dependent on personal characteristics, but not where people live. Loneliness may also result from social isolation, and may function as a mediator or moderator between social isolation and health outcomes. Examining these relationships was beyond the scope of the present paper, but should be examined in future research.

The present study is not without limitations. Although a wide range of measures regarding social networks are available in CLSA, contact with social network members is measured in CLSA only in terms of in-person contact. Other modes of contact (e.g., telephone, internet) with friends and families are not captured. There are also no measures in CLSA that assess the quality of relationships. As such, it is possible that a person with a very a small social network may in fact have sufficient emotional support, whereas an individual with a large social network may lack support. Moreover, the data are cross-sectional in nature and, as such, it cannot be inferred that the personal and geographic factors examined here predict social isolation and loneliness. For example, it is equally plausible that social isolation and loneliness are risk factors for developing health problems [1–9] and individuals with certain characteristics may self-select into certain geographic areas. The data may also exclude the most socially isolated and lonely individuals, as they may not participate in research. Our geographic unit of analyses were FSAs. Although they are meaningful in terms of defining areas of residence, some FSAs are large. Examining socio-demographic characteristics at a smaller geographic scale could provide a more detailed assessment of geographic correlates of social isolation and loneliness. Lastly, “urban” and “rural” are varyingly defined in Canada [73]. Using a different definition might have yielded different results. For example, differentiating between degrees of rurality (e.g., far north and more southern) may be useful in future research, given the very remote areas that exist in Canada. Different results might also have been obtained if we had been able to use a rural/urban definition at the FSA level in the analyses, consistent with the other geographic variables in this study.

In conclusion, the present study adds to the literature by examining not only personal factors associated with social isolation and loneliness, but also geographic factors. A strength of the study is that it was based on a national sample of Canadians aged 45 to 85. The findings may assist researchers and policy makers in identifying individuals at risk of social isolation and loneliness. They also provide a foundation for further researcher once follow-up data become available, such as a more in depth analysis of the role of low-income neighborhoods in social isolation and loneliness, an examination of the relationship between social isolation and loneliness and physical and mental health problems, and the potential mediational or moderating role of loneliness in the relationship between social isolation and health outcomes.
